# Supplementary figures and images for: The nuclear egress complex of Epstein-Barr virus buds membranes through an oligomerization-driven mechanism
Source: PLoS Pathog. 2022 Jul 8;18(7):e1010623. doi: 10.1371/journal.ppat.1010623 (PMC9299292; doi:10.1371/journal.ppat.1010623)

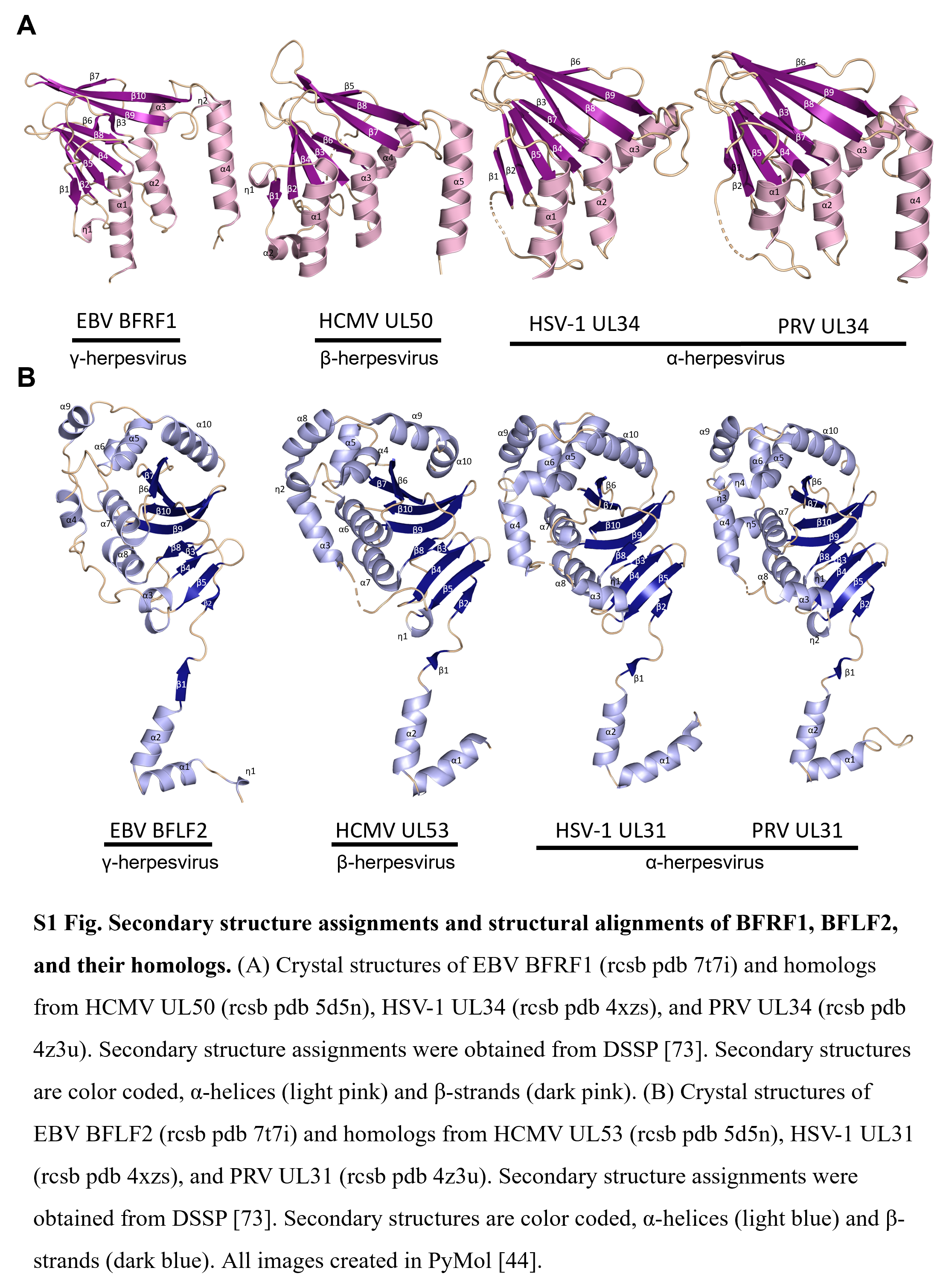

Supplement: S1 Fig — (A) Crystal structures of EBV BFRF1 (rcsb pdb 7t7i) and homologs from HCMV UL50 (rcsb pdb 5d5n), HSV-1 UL34 (rcsb pdb 4xzs), and PRV UL34 (rcsb pdb 4z3u). Secondary structure assignments were obtained from DSSP [73]. Secondary structures are color coded, α-helices (light pink) and β-strands (dark pink). (B) Crystal structures of EBV BFLF2 (rcsb pdb 7t7i) and homologs from HCMV UL53 (rcsb pdb 5d5n), HSV-1 UL31 (rcsb pdb 4xzs), and PRV UL31 (rcsb pdb 4z3u). Secondary structure assignments were obtained from DSSP [73]. Secondary structures are color coded, α-helices (light blue) and β-strands (dark blue). All images created in PyMol [44]. (TIF) [file ppat.1010623.s001.tif]

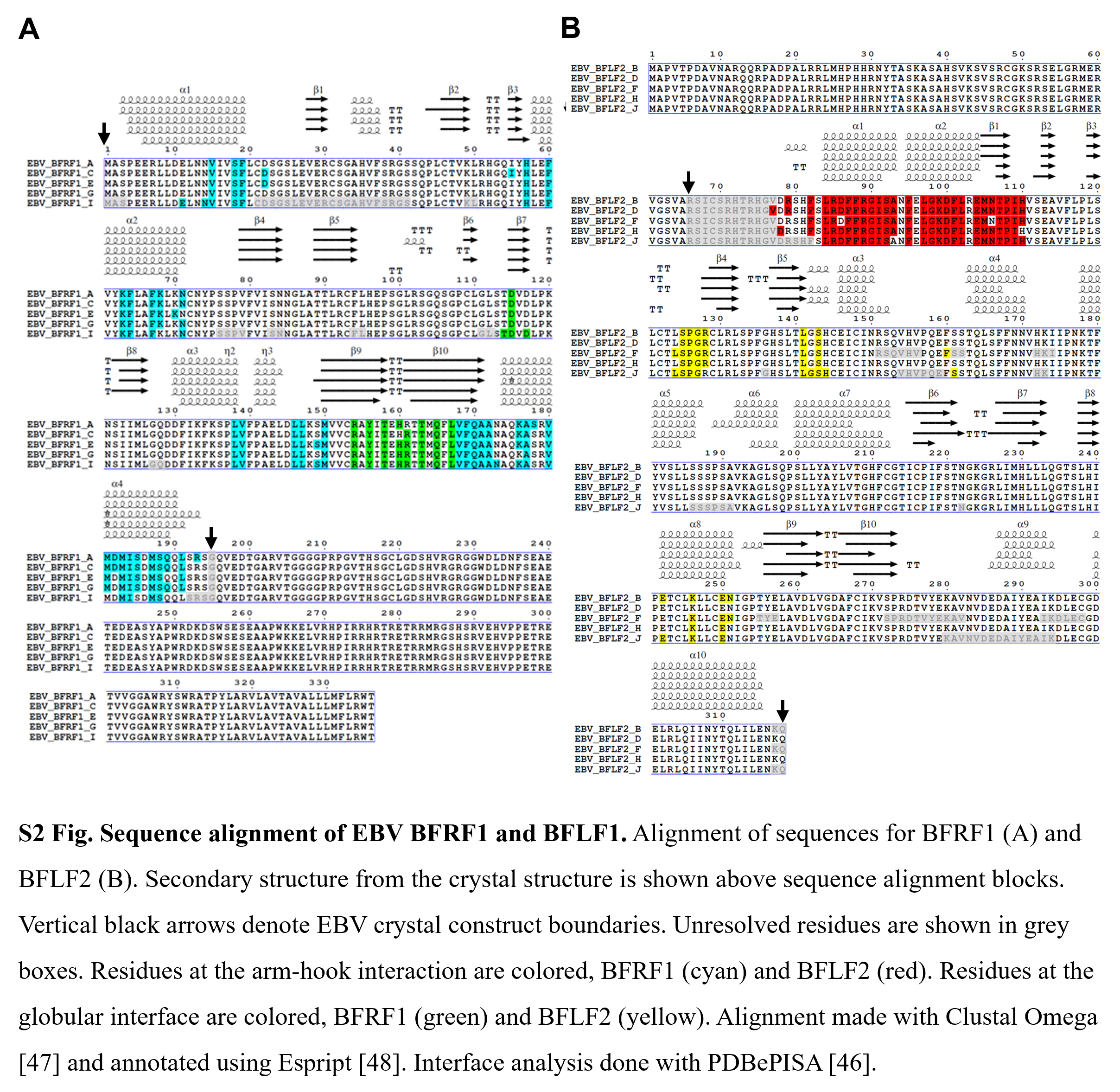

Supplement: S2 Fig — Alignment of sequences for BFRF1 (A) and BFLF2 (B). Secondary structure from the crystal structure is shown above sequence alignment blocks. Vertical black arrows denote EBV crystal construct boundaries. Unresolved residues are shown in grey boxes. Residues at the arm-hook interaction are colored, BFRF1 (cyan) and BFLF2 (red). Residues at the globular interface are colored, BFRF1 (green) and BFLF2 (yellow). Alignment made with Clustal Omega [47] and annotated using Espript [48]. Interface analysis done with PDBePISA [46]. (TIF) [file ppat.1010623.s002.tif]

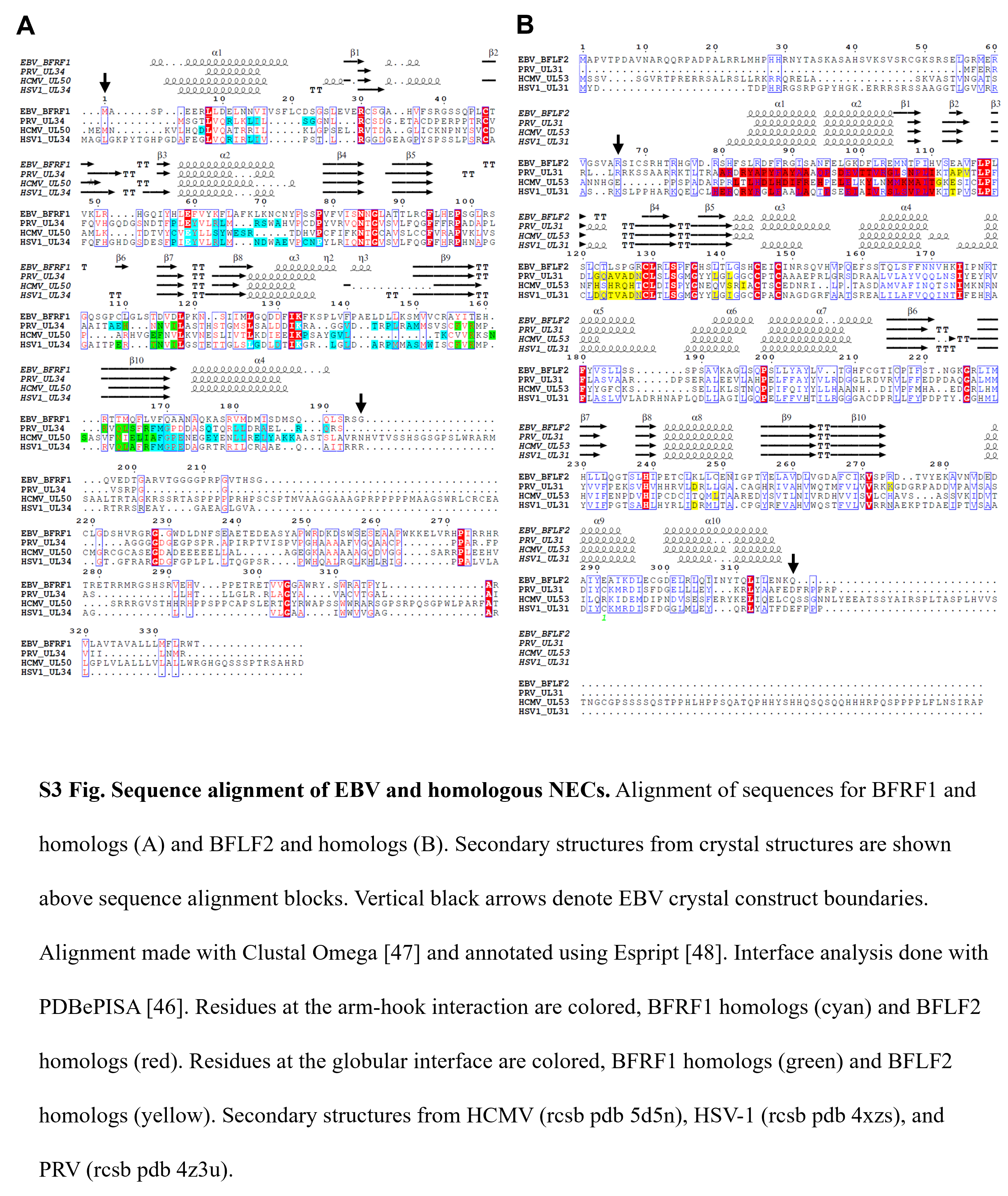

Supplement: S3 Fig — Alignment of sequences for BFRF1 and homologs (A) and BFLF2 and homologs (B). Secondary structures from crystal structures are shown above sequence alignment blocks. Vertical black arrows denote EBV crystal construct boundaries. Alignment made with Clustal Omega [47] and annotated using Espript [48]. Interface analysis done with PDBePISA [46]. Residues at the arm-hook interaction are colored, BFRF1 homologs (cyan) and BFLF2 homologs (red). Residues at the globular interface are colored, BFRF1 homologs (green) and BFLF2 homologs (yellow). Secondary structures from HCMV (rcsb pdb 5d5n), HSV-1 (rcsb pdb 4xzs), and PRV (rcsb pdb 4z3u). (TIF) [file ppat.1010623.s003.tif]
