# Supplementary material for: The nuclear egress complex of Epstein-Barr virus buds membranes through an oligomerization-driven mechanism
Source: PLoS Pathog. 2022 Jul 8;18(7):e1010623. doi: 10.1371/journal.ppat.1010623 (PMC9299292; doi:10.1371/journal.ppat.1010623)
Supplement: S1 Table — For each EBV chain, the boundaries of resolved residues and % resolved residues are listed. (DOCX) [file ppat.1010623.s004.docx]

| Chain ID | Resolved Residues | | % Resolved Residues |
| --- | --- | --- | --- |
|  | | **BFRF1** | **Total: 195 residues** |
| A | 1-194 | | 99 |
| C | 1-194 | | 99 |
| E | 1-194 | | 99 |
| G | 1-194 | | 99 |
| I | 4-20, 42-48, 51-75, 80-82, 85-93, 96-110, 113-126, 129-191 | | 78 |
|  | | **BFLF2** | **Total: 253 residues** |
| B | 78-316 | | 89 |
| D | 77-318 | | 91 |
| F | 78-150, 157-160, 163-171, 175-254, 258-271, 283-292, 299-316 | | 77 |
| H | 78-318 | | 91 |
| J | 83-135, 137-153, 160-171, 174-185, 192-221, 223-279, 295-316 | | 75 |

**S1 Table. Resolved residues for each individual EBV BFRF1 (top) and BFLF2 (bottom) crystal structure chains.** For each EBV chain, the boundaries of resolved residues and % resolved residues are listed.
