# Supplementary material for: The nuclear egress complex of Epstein-Barr virus buds membranes through an oligomerization-driven mechanism
Source: PLoS Pathog. 2022 Jul 8;18(7):e1010623. doi: 10.1371/journal.ppat.1010623 (PMC9299292; doi:10.1371/journal.ppat.1010623)
Supplement: S2 Table — Heterodimers (NEC) and individual BFRF1 or BFLF2 chains were aligned. For each EBV NEC or chain, RMSD (Å) is listed followed by the number of aligned residues in parentheses. In all cases, “SSM superpose” in WinCoot [66] was used to carry out the structure alignments and calculate RMSDs, except for those denoted by * in which case “LSQ Superpose” command was used. (DOCX) [file ppat.1010623.s005.docx]

|  | NEC1 | NEC2 | NEC3 | NEC4 | NEC5 |
| --- | --- | --- | --- | --- | --- |
| NEC1 | - | 2.57 (388) | 1.38 (387) | 1.70 (414) | 1.16 (350) |
| NEC2 | 2.57 (388) | - | 2.52 (353) | 1.92 (420) | 2.41 (337) |
| NEC3 | 1.38 (387) | 2.52 (353) | - | 1.73 (376) | 1.53 (329) |
| NEC4 | 1.70 (414) | 1.92 (420) | 1.73 (376) | - | 1.61 (353) |
| NEC5 | 1.16 (350) | 2.41 (337) | 1.53 (329) | 1.61 (353) | - |

| **BFRF1** | Chain A | Chain C | Chain E | Chain G | Chain I |
| --- | --- | --- | --- | --- | --- |
| Chain A | - | 0.76 (191) | 0.79 (186) | 0.75 (189) | 1.01 (150) |
| Chain C | 0.76 (191) | - | 0.73 (185) | 0.79 (191) | 1.14 (150) |
| Chain E | 0.79 (186) | 0.73 (185) | - | 0.72 (191) | 1.04 (151) |
| Chain G | 0.75 (189) | 0.79 (191) | 0.72 (191) | - | 1.05 (149) |
| Chain I | 1.01 (150) | 1.14 (150) | 1.04 (151) | 1.05 (149) | - |
| **BFLF2** | Chain B | Chain D | Chain F | Chain H | Chain J |
| Chain B | - | 1.81 (215) | 1.45 (198) | 1.66 (231) | 1.25 (201) |
| Chain D | 1.81 (215) | - | 2.41 (187) | 1.77 (234) | 2.16 (198) |
| Chain F | 1.45 (198) | 2.41 (187) | - | 1.64 (193) | 1.45 (177) |
| Chain H | 1.66 (231) | 1.77 (234) | 1.64 (193) | - | 1.46 (201) |
| Chain J | 1.25 (201) | 2.16 (198) | 1.45 (177) | 1.46 (201) | - |

**S2 Table. Structural alignments of the five EBV NEC heterodimers in the asymmetric unit.** Heterodimers (NEC) and individual BFRF1 or BFLF2 chains were aligned. For each EBV NEC or chain, RMSD (Å) is listed followed by the number of aligned residues in parentheses. In all cases, “SSM superpose” in WinCoot [1] was used to carry out the structure alignments and calculate RMSDs, except for those denoted by * in which case “LSQ Superpose” command was used.

**Reference**

1. Emsley, P., et al., *Features and development of Coot.* Acta crystallographica. Section D, Biological crystallography, 2010. **66**(Pt 4): p. 486-501.
