# Supplementary material for: The nuclear egress complex of Epstein-Barr virus buds membranes through an oligomerization-driven mechanism
Source: PLoS Pathog. 2022 Jul 8;18(7):e1010623. doi: 10.1371/journal.ppat.1010623 (PMC9299292; doi:10.1371/journal.ppat.1010623)
Supplement: S3 Table — Clustal Omega [47] was used for sequence alignment and calculations of % identity. The following UniProtKB IDs were used: HSV-1 UL34 (P10218), HSV-1 UL31 (P10215), PRV UL34 (G3G8R3), PRV UL31 (G3G955), HCMV UL50 (P16791), HCMV UL53 (P16794), EBV BFRF1 (P03185), and EBV BFLF2 (P0CK47). (DOCX) [file ppat.1010623.s006.docx]

|  | ***α*-herpesviruses** | | ***β*-herpesvirus** | ***γ*-herpesvirus** |
| --- | --- | --- | --- | --- |
|  | HSV-1 UL34 | PRV UL34 | HCMV UL50 | EBV BFRF1 |
| HSV-1 UL34 | - | 52% | 24% | 20% |
| PRV UL34 | 52% | - | 21% | 21% |
| HCMV UL50 | 24% | 21% | - | 20% |
| EBV BFRF1 | 20% | 21% | 20% | - |
|  | HSV-1 UL31 | PRV UL31 | HCMV UL53 | EBV BFLF2 |
| HSV-1 UL31 | - | 54% | 18% | 19% |
| PRV UL31 | 54% | - | 15% | 21% |
| HCMV UL53 | 18% | 15% | - | 24% |
| EBV BFLF2 | 19% | 21% | 24% | - |

**S3 Table. Sequence conservation among homologous herpesvirus NEC components.** Clustal Omega [1] was used for sequence alignment and calculations of % identity. The following UniProtKB IDs were used: HSV-1 UL34 (P10218), HSV-1 UL31 (P10215), PRV UL34 (G3G8R3), PRV UL31 (G3G955), HCMV UL50 (P16791), HCMV UL53 (P16794), EBV BFRF1 (P03185), and EBV BFLF2 (P0CK47).

References

1. Madeira, F., et al., The EMBL-EBI search and sequence analysis tools APIs in 2019. Nucleic acids research, 2019. 47(W1): p. W636-W641.
