# Supplementary material for: The nuclear egress complex of Epstein-Barr virus buds membranes through an oligomerization-driven mechanism
Source: PLoS Pathog. 2022 Jul 8;18(7):e1010623. doi: 10.1371/journal.ppat.1010623 (PMC9299292; doi:10.1371/journal.ppat.1010623)
Supplement: S4 Table — For each EBV NEC or chain, RMSD (Å) is listed followed by the number of residues aligned in parentheses. In all cases, “SSM Superpose” in WinCoot [66] was used to carry out the structure alignments and calculate RMSDs, except for those denoted by * in which case “LSQ Superpose” command was used. RMSD and residues aligned comparisons for BFLF2 chains B-J are not available for EBV due to the presence of only 32 residues of BFLF2 in rcsb pdb 6t3z [42]. Crystal structures of EBV BFRF1 (rcsb pdb 6t3z) and NEC homologs from HSV-1 (rcsb pdb 4xzs), PRV (rcsb pdb 4z3u and 5e8c), and HCMV (rcsb pdb 5d5n and 5dob) were used. (DOCX) [file ppat.1010623.s007.docx]

| **NEC** |  | HSV-1  NEC | PRV  NEC | PRV  NEC | HCMV NEC | HCMV NEC |
| --- | --- | --- | --- | --- | --- | --- |
|  |  | 4zxs | 4z3u | 5e8c | 5d5n | 5dob |
| NEC1 |  | 3.45 (324) | 3.23 (360) | 3.00 (207) | 3.83 (329) | 3.38 (249) |
| NEC2 |  | 3.77 (339) | 3.40 (348) | 3.66 (237) | 3.17 (341) | 3.73 (357) |
| NEC3 |  | 3.76 (299) | 3.21 (331) | 3.20 (316) | 3.66 (274) | 2.64 (253) |
| NEC4 |  | 3.03 (320) | 2.71 (357) | 3.75 (293) | 3.41 (338) | 3.34 (242) |
| NEC5 |  | 3.38 (278) | 3.08 (306) | 3.41 (181) | 3.57 (282) | 3.55 (202) |

| **BFRF1** | EBV BFRF1 | HSV-1 UL34 | PRV UL34 | PRV UL34 | HCMV UL50 | HCMV UL50 |
| --- | --- | --- | --- | --- | --- | --- |
| Chain A | 0.65 (183) | 2.08 (138) | 2.36 (149) | 2.31 (147) | 1.79 (152) | 1.69 (150) |
| Chain C | 0.64 (183) | 2.02 (140) | 2.31 (148) | 2.19 (141) | 1.91 (151) | 1.71 (150) |
| Chain E | 0.59 (183) | 2.07 (138) | 2.24 (148) | 2.33 (147) | 1.73 (145) | 1.59 (151) |
| Chain G | 0.59 (183) | 2.04 (140) | 2.21 (145) | 2.18 (143) | 1.78 (149) | 1.68 (153) |
| Chain I | 1.03 (144) | 1.95 (115) | 2.28 (123) | 2.31 (121) | 1.75 (122) | 1.63 (124) |
| **BFLF2** |  | HSV-1 UL31 | PRV UL31 | PRV UL31 | HCMV UL53 | HCMV UL53 |
| Chain B |  | 2.59 (212) | 2.66 (222) | 2.58 (207) | 2.37 (197) | 2.99 (209) |
| Chain D |  | 2.47 (198) | 2.69 (210) | 2.80 (195) | 1.94 (204) | 2.24 (210) |
| Chain F |  | 2.44 (187) | 2.62 (192) | 2.52 (185) | 2.22 (159) | 3.26 (177) |
| Chain H |  | 2.09 (205) | 2.33 (222) | 2.27 (205) | 1.93 (199) | 2.61 (207) |
| Chain J |  | 2.37 (181) | 2.68 (196) | 2.52 (184) | 2.31 (171) | 3.01 (175) |

**S4 Table. Structural alignments of the EBV NEC to its homologs in HSV-1, PRV, and HCMV.** For each EBV NEC or chain, RMSD (Å) is listed followed by the number of residues aligned in parentheses. In all cases, “SSM Superpose” in WinCoot [1] was used to carry out the structure alignments and calculate RMSDs, except for those denoted by * in which case “LSQ Superpose” command was used. RMSD and residues aligned comparisons for BFLF2 chains B-J are not available for EBV due to the presence of only 32 residues of BFLF2 in rcsb pdb 6t3z [2]. Crystal structures of EBV BFRF1 (rcsb pdb 6t3z) and NEC homologs from HSV-1 (rcsb pdb 4xzs), PRV (rcsb pdb 4z3u and 5e8c), and HCMV (rcsb pdb 5d5n and 5dob) were used.

**References**

1. Emsley, P., et al., *Features and development of Coot.* Acta crystallographica. Section D, Biological crystallography, 2010. **66**(Pt 4): p. 486-501.

2. Muller, Y.A., et al., *High-resolution crystal structures of two prototypical beta- and gamma-herpesviral nuclear egress complexes unravel the determinants of subfamily specificity.* J Biol Chem, 2020.
