# Supplementary material for: The nuclear egress complex of Epstein-Barr virus buds membranes through an oligomerization-driven mechanism
Source: PLoS Pathog. 2022 Jul 8;18(7):e1010623. doi: 10.1371/journal.ppat.1010623 (PMC9299292; doi:10.1371/journal.ppat.1010623)
Supplement: S5 Table — PDBePISA [46] analysis of the interface area between the globular domains, hook-in-groove and total heterodimeric interface. The globular interface area was obtained by deleting the arm-hook portion of BFLF2/HSV-1 UL31/PRV UL31/HCMV UL53 from files before analysis ending at V111/K88/K55/G88, respectively. The arm-hook interaction was obtained by subtracting the globular interface area from the total heterodimeric interaction. Total heterodimeric interface area was obtained by inputting the entire crystal structure. Crystal structures the NECs homologs from HSV-1 (rcsb pdb 4xzs), PRV (rcsb pdb 4z3u and 5e8c), and HCMV (rcsb pdb 5d5n and 5dob) were used. (DOCX) [file ppat.1010623.s008.docx]

| Chain | Globular  Interface Area (Å2) | Hook-in-groove  Interface Area (Å2) | Total Heterodimeric  Interface area (Å2) |
| --- | --- | --- | --- |
| EBV 7t7i | | | |
| A | 390 | 1,727 | 2,117 |
| B |  |  |  |
| C | 333 | 1,674 | 2,007 |
| D |  |  |  |
| E | 409 | 1,506 | 1,915 |
| F |  |  |  |
| G | 372 | 1,574 | 1,946 |
| H |  |  |  |
| I | 464 | 1,403 | 1,867 |
| J |  |  |  |
| HSV-1 4zxs | | | |
| A | 441 | 1,325 | 1,766 |
| B |  |  |  |
| C | 429 | 1,318 | 1,747 |
| D |  |  |  |
| PRV 4z3u | | | |
| A | 439 | 1,453 | 1,892 |
| B |  |  |  |
| C | 375 | 1,462 | 1,836 |
| D |  |  |  |
| PRV 5e8c | | | |
| A | 424 | 1,380 | 1,803 |
| B |  |  |  |
| HCMV 5d5n | | | |
| A | 418 | 1,375 | 1,793 |
| B |  |  |  |
| HCMV 5dob | | | |
| A | 381 | 1,567 | 1,948 |
| B |  |  |  |

**S5 Table. Interface areas for the globular, hook-in-groove, and total heterodimeric interfaces for the EBV BFLF2:BFRF1 interaction.** PDBePISA [1] analysis of the interface area between the globular domains, hook-in-groove and total heterodimeric interface. The globular interface area was obtained by deleting the arm-hook portion of BFLF2/HSV-1 UL31/PRV UL31/HCMV UL53 from files before analysis ending at V111/K88/K55/G88, respectively. The arm-hook interaction was obtained by subtracting the globular interface area from the total heterodimeric interaction. Total heterodimeric interface area was obtained by inputting the entire crystal structure. Crystal structures the NECs homologs from HSV-1 (rcsb pdb 4xzs), PRV (rcsb pdb 4z3u and 5e8c), and HCMV (rcsb pdb 5d5n and 5dob) were used.

**Reference**

1. Krissinel, E. and K. Henrick, *Inference of macromolecular assemblies from crystalline state.* J Mol Biol, 2007. **372**(3): p. 774-97.
