# Supplementary material for: The nuclear egress complex of Epstein-Barr virus buds membranes through an oligomerization-driven mechanism
Source: PLoS Pathog. 2022 Jul 8;18(7):e1010623. doi: 10.1371/journal.ppat.1010623 (PMC9299292; doi:10.1371/journal.ppat.1010623)
Supplement: S6 Table — PDBePISA [46] analysis of the solvent accessible area buried at the oligomeric interface. Crystal structures of the NEC homologs from HSV-1 (rcsb pdb 4xzs) and HCMV (rcsb pdb 5d5n) were used. (DOCX) [file ppat.1010623.s009.docx]

| Chain | Oligomeric Interface area (Å^2^) |
| --- | --- |
|  |  |
| EBV 7t7i | |
| A | 759 |
| H |  |
| C | 967 |
| F |  |
| HSV-1 4zxs | |
| A | 613 |
| B |  |
| C | 572 |
| D |  |
| HCMV 5d5n | |
| A | 760 |
| B |  |

**S6 Table. Interface areas for the oligomeric interfaces of EBV NEC and all applicable homologous NEC crystal structures.** PDBePISA [1] analysis of the solvent accessible area buried at the hexameric interface. Crystal structures of the NEC homologs from HSV-1 (rcsb pdb 4xzs) and HCMV (rcsb pdb 5d5n) were used.

**Reference**

1. Krissinel, E. and K. Henrick, *Inference of macromolecular assemblies from crystalline state.* J Mol Biol, 2007. **372**(3): p. 774-97.
