# Supplementary material for: The nuclear egress complex of Epstein-Barr virus buds membranes through an oligomerization-driven mechanism
Source: PLoS Pathog. 2022 Jul 8;18(7):e1010623. doi: 10.1371/journal.ppat.1010623 (PMC9299292; doi:10.1371/journal.ppat.1010623)
Supplement: S7 Table — Interface residues in the two EBV NEC dimers in the crystal structure were mapped using PDBePISA [46] analysis (Fig 4). Interface residues that are identical across in BFLF2 and its homologs or BFRF1 and its homologs from EBV, HSV-1, and HCMV (Identical interface residues) were divided by the total number of interface residues in the respective dimer (Total interface residues) to yield % Identical Interface Residues. % Total Sequence Conservation is the % identical residues in BFLF2 or BFRF1. (DOCX) [file ppat.1010623.s010.docx]

|  | Identical Interface Residues | Total Interface Residues | % Identical Interface Residues | % Total Sequence Conservation |
| --- | --- | --- | --- | --- |
| Chain F (BFLF2) | 2 | 18 | 11% | 7% |
| Chain C (BFRF1) | 1 | 26 | 4% | 6% |
|  |  |  |  |  |
| Chain H (BFLF2) | 3 | 15 | 20% | 7% |
| Chain A (BFRF1) | 0 | 17 | 0% | 6% |

**S7 Table. Conservation of residues at the oligomeric interfaces.** Interface residues in the two EBV NEC dimers in the crystal structure were mapped using PDBePISA [1] analysis (Fig. 4). Interface residues that are identical across in BFLF2 and its homologs or BFRF1 and its homologs from EBV, HSV-1, and HCMV (Identical interface residues) were divided by the total number of interface residues in the respective dimer (Total interface residues) to yield % Identical Interface Residues. % Total Sequence Conservation is the % identical residues in BFLF2 or BFRF1.

**References**

1. Krissinel, E. and K. Henrick, *Inference of macromolecular assemblies from crystalline state.* J Mol Biol, 2007. **372**(3): p. 774-97.
