# Supplementary material for: The nuclear egress complex of Epstein-Barr virus buds membranes through an oligomerization-driven mechanism
Source: PLoS Pathog. 2022 Jul 8;18(7):e1010623. doi: 10.1371/journal.ppat.1010623 (PMC9299292; doi:10.1371/journal.ppat.1010623)
Supplement: S8 Table — Mutations are bolded, restriction digest sites are underlined and listed underneath applicable primers. BFRF1 constructs are codon optimized (c.o.). (DOCX) [file ppat.1010623.s011.docx]

| **Plasmid** | **Construct Info** | **Forward Primer** | **Reverse Primer** |
| --- | --- | --- | --- |
| pJB75 | EBV BFLF2 66-318 | 5’-aaaaaaggatcccgctcaatatgttcccggcac-3’  BamHI | 5’-aaaaaaaagcttttactgtttattttccaaaatgagctgggtatag-3’  HindIII |
| pJB77 | EBV BFRF1 1-195 c.o. | 5’-aaaaaaggatccatggcaagtccggaag-3’  BamHI | 5’-aaaaaagaattctcaaccactacggctcagctgctg-3’  EcoRI |
| pJB73 | EBV BFRF1 1-228 c.o. | 5’-aaaaaaggatccatggcaagtccggaag-3’  BamHI | 5’-aaaaaagaattcttaacgaccacgaacatggctatcac-3’  EcoRI |
| pMT05 | EBV BLFL2 1-318 | 5’-aaaaaaggatccatggccccggtcacccc-3’  BamHI | 5’-aaaaaagaattcttagcgccccctaacgtgtgag-3’  EcoRI |
| pMT37 | EBV BFLF2 16-318 | 5'-ggatccgggcccctggaac-3' | 5'-ggatccgggcccctggaac-3'  BamHI |
| pMT38 | EBV BFLF2 25-318 | 5'-atgcatccgcaccacagaaactacac-3' | 5'-ggatccgggcccctggaac-3'  BamHI |
| pMT39 | EBV BFLF2 35-318 | 5'-tcaaaggcctcggcgcatagcg-3' | 5'-ggatccgggcccctggaac-3'  BamHI |
| pMT40 | EBV BFLF2 45-318 | 5'-gtgtccaggtgtggaaaatctcgc-3' | 5'-ggatccgggcccctggaac-3'  BamHI |
| pMT46 | EBV BFRF1 215-His c.o. | 5'-cgtccgagcgttaccgggcggccgcaccacca-3'  NotI | 5'-cggaccaccaccacctgtaacacgtgcacctgtatctt-3' |
| pMT47 | EBV BFRF1 228-His c.o. | 5'-aaaaaaggatccatggcaagtccggaag-3'  BamHI | 5'-aaaaaagcggccgcccggtaacgctcggacgcggac-3'  NotI |
| pMT49 | EBV BLFL2 N31S 1-318 | 5'-cgcaccacaga**agt**tacacggcc-3' | 5'-ggccgtgta**act**tctgtggtgcg-3' |
| pMT54 | EBV BFRF1 1-215 L87W c.o. | 5'-agcaataatggt**tgg**gcaaccaccctg-3' | 5'-cagggtggttgc**cca**accattattgct-3' |
| pMT56 | EBV BFRF1 1-215 Q43A c.o. | 5'-cgtggtagcagc**gcg**ccgctgtgtacc-3' | 5'-ggtacacagcgg**cgc**gctgctaccacg-3' |
| pMT57 | EBV BFRF1 1-215 N121Q c.o. | 5'-gatctgccgaaa**cag**agcattattatg-3' | 5'-cataataatgct**ctg**tttcggcagatc-3' |
| pMT59 | EBV BFLF2 F.L. N31S L262F | 5'-tggccgtggac**ttt**gtaggggacgc-3' | 5'-gcgtcccctac**aaa**gtccacggcca-3' |
| pMT62 | EBV BFLF2 F.L. N31S F267W | 5'-gtaggggacgcc**tgg**tgcataaaggtc-3' | 5'-gacctttatgca**cca**ggcgtcccctac-3' |

**S9 Table.** **List of primers used for cloning described in Materials and Methods.** Mutations are bolded, restriction digest sites are underlined and listed underneath applicable primers. BFRF1 constructs are codon optimized (c.o.).
