# Supplementary material for: The nuclear egress complex of Epstein-Barr virus buds membranes through an oligomerization-driven mechanism
Source: PLoS Pathog. 2022 Jul 8;18(7):e1010623. doi: 10.1371/journal.ppat.1010623 (PMC9299292; doi:10.1371/journal.ppat.1010623)
Supplement: S9 Table — Due to different isoelectric points (PIs) of EBV NEC constructs, pH of buffers used for purification was adjusted to be ~1 pH unit away from the pI. Cells were lysed in lysis buffer (50 mM indicated buffer, 500 mM NaCl, 0.5 mM TCEP, 10% glycerol). Affinity tag removal changed the PIs, so buffer with a different pH was used during and after PreScission protease cleavage. After affinity tag removal, protein was further purified in gel filtration buffer (20 mM indicated buffer, 100 mM NaCl, 0.5 mM TCEP). Salt concentration varied for ion exchange chromatography (see Materials and Methods). (DOCX) [file ppat.1010623.s012.docx]

|  | **pI** | | **Buffer pH (50 mM for lysis, 20 mM for gel filtration)** | | |
| --- | --- | --- | --- | --- | --- |
| **EBV NEC Construct** | **Before**  **PreScission**  **cut** | **After**  **PreScission**  **cut** | **Lysis Buffer Before PreScission cut** | **Lysis Buffer During PreScission Cut** | **Gel Filtration Buffer** |
| 195Δ65 | 6.39 | 7.29 | HEPES pH 7.5 | HEPES pH 7.5 | HEPES pH 8 |
| 228Δ44-His_8_ | 6.84 | 8.34 | HEPES pH 8 | HEPES pH 8 | HEPES pH 7 |
| 228Δ34-His_8_ | 7.00 | 8.53 | HEPES pH 8 | HEPES pH 8 | HEPES pH 7 |
| 228Δ24-His_8_ | 7.17 | 8.61 | Tris pH 8.5 (@ 4°C) | MES pH 5.5 | HEPES pH 7.5 |
| 228Δ15-His_­8_ | 7.3 | 8.69 | MES pH 6 | MES pH 6 | HEPES pH 7 |
| 215-N31S_BFLF2_-His_8_ | 6.61 | 7.49 | Tris pH 9 (@ 4°C) | Tris pH 8.5 (@ 4°C) | Tris pH 8.5 (@ 4°C) |
| 215-L87W_BFRF1_/N31S_BFLF2_-His_8_ | 6.61 | 7.49 | Tris pH 9 (@ 4°C) | Tris pH 8.5 (@ 4°C) | Tris pH 8.5 (@ 4°C) |
| 215-Q43A_BFRF1_/N31S_BFLF2_-His_8_ | 6.61 | 7.49 | Tris pH 9 (@ 4°C) | Tris pH 8.5 (@ 4°C) | Tris pH 8.5 (@ 4°C) |
| 215-N121Q_BFRF1_/N31S_BFLF2_-His_8_ | 6.61 | 7.49 | Tris pH 9 (@ 4°C) | Tris pH 8.5 (@ 4°C) | Tris pH 8.5 (@ 4°C) |
| 215-N31S_BFLF2_/L262F_BFLF2_-His_8_ | 6.61 | 7.49 | Tris pH 9 (@ 4°C) | Tris pH 8.5 (@ 4°C) | Tris pH 8.5 (@ 4°C) |
| 215-N31S_BFLF2_/F267W_BFLF2_-His_8_ | 6.61 | 7.49 | Tris pH 9 (@ 4°C) | Tris pH 8.5 (@ 4°C) | Tris pH 8.5 (@ 4°C) |
| 215-N31S_BFLF2_ | 6.5 | 7.39 | HEPES pH 7.5 | MES pH 6 | MES pH 6 |
| **HSV-1 NEC Construct** |  |  |  |  |  |
| 220 | 6.94 | 8.7 | HEPES pH 7.5 | HEPES pH 7.5 | HEPES pH 7.0 |

**S9 Table. Protein purification buffers.** Due to different isoelectric points (PIs) of EBV NEC constructs, pH of buffers used for purification was adjusted to be ~1 pH unit away from the pI. Cells were lysed in lysis buffer (50 mM indicated buffer, 500 mM NaCl, 0.5 mM TCEP, 10% glycerol). Affinity tag removal changed the PIs, so buffer with a different pH was used during and after PreScission protease cleavage. After affinity tag removal, protein was further purified in gel filtration buffer (20 mM indicated buffer, 100 mM NaCl, 0.5 mM TCEP). Salt concentration varied for ion exchange chromatography (see Materials and Methods).
