# Supplementary material for: The nuclear egress complex of Epstein-Barr virus buds membranes through an oligomerization-driven mechanism
Source: PLoS Pathog. 2022 Jul 8;18(7):e1010623. doi: 10.1371/journal.ppat.1010623 (PMC9299292; doi:10.1371/journal.ppat.1010623)
Supplement: Attachment — Submitted filename: Thorsen-NEC-PLOSPath-response-to-criticisms-04252022.docx [file ppat.1010623.s013.docx]

**Re: PPATHOGENS-D-21-02596**

**The nuclear egress complex of Epstein-Barr virus buds membranes through a distinct, oligomerization-driven mechanism**

We are grateful for the reviewers' thoughtful suggestions and criticisms and for the opportunity to submit the revised version of the manuscript. Our point-by-point response is provided below. For convenience, we provide both the “clean” and the tracked versions of the revised manuscript. Changes in response to reviewers’ criticisms are indicated in blue font in the “clean” version.

**Reviewer Comments:**

**Reviewer #1:**

Thorsen and Heldwein have determined the structure of BFRF1 in complex with BFLF2 - the nuclear egress complex (NEC) from EBV. Despite considerable variation in amino acid sequences between EBV NEC components and NEC proteins from HSV-1, PRV and HCMV the structure of the EBV NEC appears very similar to the alpha- and betaherpesvirus NECs suggesting the gammaherpesviruses utilize a similar mechanism for primary envelopment of capsids at the inner nuclear membrane. While a hexagonal lattice of NEC components was observed in cryo-ET reconstructions of the HSV-1 NEC as well as in the NEC crystals formed by HSV-1 and HCMV NEC components, no hexagonal lattice was observed in the EBV NEC crystals, nor was it investigated in in vitro membrane budding assays as done in the past by this group for the HSV-1 NEC. Despite these apparent differences, NEC/NEC interaction interfaces were identified in the EBV NEC crystals that resembled those formed between NEC/NEC heterodimer pairs in HSV-1 and HCMV NEC hexamers. Mutation of amino acids residues within the putative EBV NEC/NEC interface prevented membrane budding in an in vitro budding assay. The authors’ central conclusions are that the EBV NEC oligomerizes and that the putative oligomerization interface identified is required for budding. The authors further suggest that this study supports the idea that NECs from three subfamilies of the Herpesviridae operate through a common mechanism driven by the formation of a hexagonal protein lattice. Unfortunately, the data presented fail to support these conclusions. While it is appreciated that the work described is important and needed to be done to "close the loop" on NEC structural determinations of alpha-, beta- and gammaherpesviruses, the findings do not move our understanding of NEC form, or function, significantly forward.

**Response:** We thank the reviewer for bringing up an important point regarding EBV NEC oligomerization during budding. To address this concern, we performed the budding experiments with EBV NEC and visualized them by cryo-EM side-by-side with HSV-1 NEC, which is known to oligomerize into a hexagonal lattice. We found that just as HSV-1 NEC, EBV NEC forms coats on the inner surface of the budded vesicles that are 11-nm thick and have a fence-like appearance in side views, implying a periodic arrangement (see new Fig. 7). However, top views of the EBV NEC coats lack the hexagonal geometry observed in HSV-1 NEC coats. Therefore, we hypothesize that the EBV NEC oligomerizes into coats of a different geometry.

The ability of the EBV NEC to oligomerize is supported by EBV NEC dimers observed in the crystals and by the similarities between the dimeric interfaces observed in the EBV NEC crystals and the hexameric interfaces observed for the NECs from alpha and beta-herpesviruses. Moreover, mutations engineered to disrupt this interface reduce budding to the extent similar to interface mutations in HSV-1 NEC. Putting together the crystal structure, the cryo-EM data, and mutational analysis, we propose that EBV NEC-mediated budding is driven by oligomerization into coats of distinct geometry. The detailed analysis of the EBV NEC coat geometry is the subject of future work, beyond the scope of this manuscript.

**Specific criticisms:**

Major shortcomings include the failure to demonstrate: 1) NEC oligomerization outside of a crystal; 2) that the mutations introduced into the putative oligomerization interface actually prevent NEC/NEC oligomerization; and 3) no evidence that a hexagonal lattice forms during membrane budding in vitro.

**Response:** See our response above.

Statements on lines 27-28 “….that EBV NEC oligomerizes and that the oligomeric interface is conserved across homologs and essential for budding.” is not supported by the data. This also applies to statements/conclusions on lines 123-124, 354-356 and 385-386.

**Response:** See our response above.

Line 38. In addition to mammals and birds, ICTV indicates that viruses infecting turtles and iguanas are also included within the Herpesviridae.

**Response:** We thank the reviewer for pointing out this oversight and have added “…and reptiles…’ on line 59 (changes tracked) or lines 50-51 (clean).

Line 69. Not strictly true. Rabbit skin cells support the replication of HSV-1 UL31 mutants fairly well (PMID: 15476875).

**Response:** We now mention on lines 100-103 (changes tracked) or lines 82-84 (clean) that rabbit skin cells can support HSV-1 replication in the absence of UL31.

Line 101. Are authors are referring to “primary envelopment of capsids” rather than “nuclear egress”? Nuclear egress comprises the entirety of capsid recruitment to the inner nuclear membrane through de-envelopment of primary enveloped virions at the outer nuclear membrane.

**Response:** We have modified the text on line 155 (changes tracked) or line 118 (clean) to clarify that we are referring to primary capsid envelopment.

Figure 4. Consider using a color other than yellow to highlight the tilt angles. The yellow is difficult to see.

**Response:** We have changed the color to red and made the lines thicker in Fig. 3.

Figure 5. Consider adding an example of what the budding assays look like (i.e. actual data) in addition to the table.

**Response:** We have added a diagram illustrating the budding assay and representative images to Fig. 6.

Is there any evidence of a hexagonal lattice of NEC components on these membranes as described in Bigalke et al (PMID: 24916797)?

**Response:** See our response to the first comment of the reviewer.

**Reviewer #2:**

Thorsen and Heldwein explore the structure and function of the Epstein-Barr virus (EBV) nuclear egress complex (NEC) and present data supporting the hypothesis that the NECs of herpesviruses are constitutive budding machines and that they mediate budding by a conserved mechanism requiring formation of hexamers. The simple addition of another structure to the collection of herpesvirus NEC structures might not, in itself, meet the standards for originality of this journal. However, the results of the study are very interesting primarily because of unexpected differences between the structures (plural) of the EBV NEC compared to those of other herpesviruses of the alpha and beta sub-families. Unlike its homologs, the EBV NEC is structurally heterogeneous in crystals and does not form hexamers. The structural data supporting this conclusion are compelling and suggest that the EBV NEC is more flexible than its counterparts. The authors also present strong evidence showing that the EBV NEC, like its homologs, can induce budding on synthetic membranes in vitro. They present somewhat weaker evidence that this budding is sensitive to mutations in residues homologous to those that support hexamer formation in other herpesviruses. The paper is well written and appropriately referenced.

The data in the paper are quite straightforward and there is little to argue with there. The authors are also appropriately cautious with conclusions. The data supporting the authors proposal that the EBV NEC mediates budding by hexamer formation are weak, based on the in vitro behavior of a few mutations, not all of which have the expected effect. Possible reasons for the unexpected budding activity of the L87W mutation should be addressed in the discussion.

**Response:** We agree with the reviewer that the hexamer formation by the EBV NEC was not supported by the available data. In light of the new cryo-EM data that show that the EBV NEC coats do not resemble the hexagonal HSV-1 NEC coats, we have modified our conclusions and propose that EBV NEC-mediated budding is driven by oligomerization into coats of distinct geometry.

We respectfully disagree, however, that interface mutations do not support conclusion about the importance of oligomerization in budding. Four out of five interface mutations reduce budding at least 2-fold, in a statistically significant manner, compared to five out of seven interface mutations in HSV-1 NEC as reported in Bigalke et al, 2015. Although one mutation, L87W, does not have an effect on budding, we hypothesize that residue 87 can accommodate a larger tryptophan side chain being located in a flexible loop. We have added this explanation on lines 623-624 (changes tracked) or line 448-450 (clean). We have also added a table (Fig. 8E) to compare the effectiveness of analogous interface mutations between EBV and HSV-1 NEC.

A previous publication by these authors has suggested that membrane curvature in budding results from lipid ordering induced by the aggregated membrane proximal region(s) of the NEC. Might these results be consistent with the idea that the NEC need not specifically form hexamers, but that other aggregation arrangements might serve the purpose? This might be an entirely wrong-headed suggestion, but it would be interesting if the authors provided some speculation as to how the structural flexibility of the EBV NEC might fit into their ideas about how membrane curvature is induced.

**Response:** We thank the reviewer for this idea. Indeed, our cryo-EM data show that while the EBV NEC forms membrane-bound coats, these do not resemble the hexagonal coats formed by HSV-1 NEC. Nonetheless, we think the EBV NEC oligomerizes rather than aggregates because 1) the NEC spikes form a periodic, fence-like arrangement in the coats, 2) the NEC dimerizes in the crystals, and the dimeric interfaces resemble the hexameric interfaces observed in NECs of alpha- and beta-herpesviruses, and 3) interface mutations reduce budding. Therefore, we hypothesize that EBV NEC-mediated budding is driven by oligomerization, but that EBV NEC coats have distinct geometry, potentially, due to the structural flexibility of the EBV NEC. We have modified our discussion of EBV NEC oligomerization to reflect these hypotheses on lines 27-37, 191-198, 665-671, 802-816 (changes tracked) or lines 24-29, 140-146, 485-491, 588-602 (clean).

**Reviewer #3:**

In this manuscript, Thorsen and Heldwein examine the structure and assembly of the nuclear egress complex (NEC) of Epstein-Barr Virus, which is a member of the gamma-herpesvirus family. Prior work from the Heldwein and other groups have examined the NECS from other herpesviruses from the alpha- and beta-herpesviruses, but our understanding of the structure and function of these complexes in the gamma-herpesviruses has remained incomplete. While a previous manuscript has reported the structure of a fragment of the EBV NEC, here the authors determine a more complete structure of the BFLF2/BFRF1 heterodimer, albeit at moderate resolution, providing evidence that the EBV NEC oligomerizes through similar interactions to other NECS. In addition, the authors demonstrate that mutations in the N-terminal region and at the NEC oligomerization interface interfere with NEC-mediated budding, establishing that the herpesviruses utilize a common mechanism for inducing membrane budding to traverse the nuclear membrane. This manuscript contributes to our global understanding of herpesvirus assembly mechanisms through these structural and functional studies.

**Specific criticisms:**

Given the relatively low resolution of the structures, how good is the overall fitting of the models for the 5 NEC copies?

**Response:** The overall fit of the final EBV NEC model into the electron density is very good judging by the R-free of 0.3089, which is close to the mean R-free of 129 PDB entries of similar resolution, 0.305, as reported by Polygon module within the Phenix suite.

How many residues are missing in the 5 different copies?

**Response:** The missing residues for each chain are listed in the S2 Table. The NEC copies 1-3 and the UL34 of the NEC copy 4 are best resolved. Chain A has 99% residues resolved, chain B has 89%, chain C has 99%, chain D has 91%, chain E has 99%, chain F has 77%, chain G has 99%, chain H has 91%, chain I has 78%, and chain J has 75%. We now also list them in the Materials and Methods on lines 962-967 (changes tracked) and lines 734-739 (clean) and S1 Table.

How was the modeling of side chains treated throughout the structure relative to the observed electron density?

**Response:** Side chains were modeled into the available electron density. Whenever the electron density for particular side chains was missing, the most common rotamer was used. We have added this explanation on lines 958-960 (changes tracked) or lines 730-731 (clean).

While some of the variability in the different NEC copies is mentioned in the text, it is difficult to get an overview of the heterogeneity in the structures and how these might relate to the NEC interfaces. These issues should be addressed either in the main text or methods section and perhaps in a summary supplementary figure.

**Response:** We agree and have moved several panels from the supplementary figures into Fig. 1 to better illustrate the differences between the EBV NEC heterodimers. We have also added text on lines 285-291, 307-335 (changes tracked) or lines 202-207, 214-227 (clean) to clarify structural differences. S2 and S4 Tables now also list RMSDs between the NECs, BFRF1, and BFLF2 to show variability between NEC copies.

Given the relatively low overall conservation of the NECs across herpesviruses, do the authors observe any enrichment of conserved residues at the heterodimer and hexamer/oligomer interfaces?

**Response:** We thank the reviewer for bringing up this important point. Analysis of the oligomeric or heterodimer interfaces did not reveal any notable enrichment of conserved residues. We have included new table S7 and added text on lines 409-410, 448-451 (changes tracked) or lines 301-302, 334-336 (clean).

The previous structure of the hook-and-groove interaction in the BFLF2/BFRF1 heterodimer has an interface size of 1569 Å2, while this interface in the current structures varies in size from 1,425 to 1,704 Å2. What is the effect of this variation on the overall structure of the heterodimer and what regions are varying to accommodate these changes?

**Response:** The differences in the area buried at the hook-and-groove interface is due mainly to the number of interacting residues. For example, NEC1 (Chains A+B) has 59 residues at the hook-and-groove interface and the buried area of 1,704 Å^2^ whereas NEC4 (Chains G+H) has 51 residues and the buried area of 1,601 Å^2^. The N terminus of BFLF2 exhibits the highest variability between chains. These differences do not correlate with the degree of EBV NEC tilt angle or any other structural differences within the NEC and minimally contribute to the overall structural plasticity of the EBV NEC. We have added this explanation on lines 373-375 (changes tracked) or lines 265-267 (clean).

How does this variation (along with the variations in the globular head domain interactions) contribute to or impact the potential assembly of the EBV NEC heterodimers into the proposed hexameric form? Are some of these distinct conformations more or less compatible with forming hexameric assemblies?

**Response:** The two EBV NEC dimers observed in the crystals have very similar dimerization interfaces. So, structural plasticity has a modest effect on its dimerization. Nonetheless, it could influence the EBV NEC coat formation, which we now note on lines 578-586, 664-671, 755-759, 802-816 (changes tracked) or lines 410-415, 489-491, 557-560, 588-602 (clean).

Lines 314-316: It would be helpful to the reader to briefly explain the budding assay in the main text and also to clarify in Figure 5 what ILVs are. What is the background level of ILVs in this assay relative to the specific signal?

**Response:** We have added a diagram illustrating the budding assay and representative images, with arrows indicating the ILVs, to Fig. 6. The background level of ILVs is ~10%, which has been added to the figure legends of Fig 6 and Fig 8 and line 993 (changes tracked) or line 764 (clean).

Line 309: typo “added to the C terminus of BFRF1 tether proteins”

**Response:** We have modified the text on lines 522-523 (changes tracked) or line 353 (clean) to read “added to the C terminus of BFRF1 to tether proteins”.

Line 622: Reference is made to Figure 4c, showing ILVs, but this figure was not included. I think adding this panel to one of the original figures would be useful for readers unfamiliar with the budding assay.

**Response:** We have changed the reference to Fig. 6C.
