# Supplementary material for: The nuclear egress complex of Epstein-Barr virus buds membranes through an oligomerization-driven mechanism
Source: PLoS Pathog. 2022 Jul 8;18(7):e1010623. doi: 10.1371/journal.ppat.1010623 (PMC9299292; doi:10.1371/journal.ppat.1010623)
Supplement: Attachment — Submitted filename: Thorsen-NEC-PLOSPath-response-to-criticisms-R1-05262022.docx [file ppat.1010623.s014.docx]

**Re: PPATHOGENS-D-21-02596****R1**

**The nuclear egress complex of Epstein-Barr virus buds membranes through a distinct, oligomerization-driven mechanism**

We are grateful for the reviewers’ thoughtful suggestions and criticisms and have toned down our oligomerization claims in the revised manuscript. Our point-by-point response is provided below.

**Reviewer 1**

**Summary**

This revised manuscript from Thorsen and colleagues is substantially improved over the previous submission and the new cryo-EM experiments, in particular, have led to new conclusions and have resulted in a more interesting study. Problems related to over-interpretation of the findings remain as do a few inaccuracies in the background information provided.

Response: We thank the reviewer for their appreciation of our added experiments and value their input with regard to over-interpretation and background information inaccuracies. We have addressed specific criticisms below.

**Major Issues: Key Experiments Required for Acceptance**

1) Line 426 and line 564 of the “clean” version of the MS. Despite the conclusions of these section headings, the authors have not demonstrated that NEC oligomerization is “essential” or “required” for budding and these statements represent an over-interpretation of the findings. The data clearly demonstrate NEC oligomerization occurs and that some mutations (but not others) introduced into the NEC/NEC interface that were intended to prevent oligomerization interfered with budding, however, NEC/NEC oligomerization of these mutants was not assessed. Thus, the authors cannot conclude that oligomerization is essential, or required, for budding. The authors can conclude that residues in the NEC/NEC oligomerization interface are required for optimal budding.

Response: We agree and have changed the text to state that “The NEC oligomeric interface is important for budding” (line 428) and that “The ability of the NEC to oligomerize is conserved,” (line 565).

**Minor Issues: Editorial and Data Presentation Modifications**

1) Lines 34-35 of the “clean” version of the MS. "nuclear budding" is not the first step in the process of nuclear egress. At the very least, budding/capsid envelopment is preceded by recruitment of capsids to the inner nuclear membrane as is activation of the NEC, which has been shown, by several groups, to be negatively regulated in infected cells.

Response: We agree and now refer to nuclear budding as “an early step” in herpesvirus nuclear egress (line 34).

2) Lines 56-58 of the “clean” version of the MS. This statement is misleading - e.g. it implies that KSHV infects "most of the world's population", which is not true.

Response: We have modified the text to say “Together, these viruses…” to clarify that we are referring to all viruses described above line 56 instead of only KSHV (line 56).

3) Line 788. Dr. Draganova is an author of the revised manuscript and likely does not require additional acknowledgement in the Acknowledgements section.

Response: We apologize for this oversight and have removed Dr. Draganova from the Acknowledgements section.

**Reviewer 2**

**Summary**

The revised manuscript by Thorsen et al., represents a significant improvement over the first. The newer data showing more results and a cryo-EM imaging of the results of the in vitro budding assay are particularly interesting and help raise the impact of the study. This new data, however, has some problematic aspects that are not addressed critically by the authors. Specifically, the cryo-EM images in Figure 7 do not strongly support either the authors’ descriptions of them or the conclusions they attempt to draw.

Response: We appreciate the reviewer’s comment noting the significant improvement of our manuscript. We have edited our conclusions to better reflect the new data in figure 7, as detailed below.

(i) the “fence-like” appearance of the protein coat in the side view of the EBV NEC budded vesicles can only be apprehended in a small area of the membrane of the budded vesicle shown and bears no resemblance to the side view from the HSV NEC budded vesicle shown. The spacing of the vertical elements in the EBV image is much tighter than that in the HSV image and they are much narrower. I am unconvinced that these vertical elements in the EBV image represent NEC heterodimers or even that they represent the major structural feature of the vesicle side views.

Response: We agree with the reviewer that the EBV NEC coats lack an obvious fence-like appearance in side views and have this description out. We now only state that EBV NEC coats are ~11 nm in thickness (line 399).

It is also worth noting that the clearest structural element in the EBV image shown is a set of lateral striations layered interior to the vesicle membrane on the right hand side of the vesicle, beginning just above the enlarged side view area. It’s not clear why these are not also a focus of analysis. Some quantitation of the frequency (perhaps % of membrane contour that shows these structures) would be helpful and higher resolution images would definitely strengthen their analysis.

Response: As the reviewer points out, higher resolution images and tomography, along with membrane contour analyses. CryoET reconstructions of the EBV NEC coats are the focus of our future work, beyond the scope of the current study. In the meantime, we have chosen to not speculate on the geometry of the EBV NEC coat other than state that it appears different from that of HSV-1 NEC.

(ii) The top views shown do not have the resolution to allow the authors to make any statement about the organization of NEC heterodimers. It’s clear that the organization of EBV and HSV membrane proteins is different in some way, since the HSV top view image suggests some structure and the EBV image really does not. There is, however, no more to say about this. Since hexamers are not evident even in the HSV image shown it is impossible to interpret their absence in the EBV image. For both these issues, higher resolution images would be very helpful, but at the very least a more critical discussion of the limitations of this data is necessary.

Response: The reviewer raises a fair point. The available data are insufficient to make firm claims regarding the EBV NEC coat geometry. Accordingly, we have changed the description of the EBV NEC coat geometry throughout the manuscript to state that it is different and/or unknown (e.g., line 24). We have also added the following statement with regards to the HSV-1 NEC coats to clarify that our data agree with previous findings showing the hexagonal geometry of the HSV-1 NEC coats: “Although current resolution is insufficient to discern the precise oligomeric arrangement of the HSV-1 NEC coats, we hypothesize that the HSV-1 NEC coats visualized here have hexagonal geometry because their appearance closely resembles the coats formed by the same HSV-1 NEC construct that had hexagonal geometry revealed by 3D averaging [25, 43]” (lines 408-411).

**Major Issues: Key Experiments Required for Acceptance**

No experiments are absolutely required.

**Minor Issues: Editorial and Data Presentation Modifications**

Reviewer #2: The paper is well written and data are clearly presented. Higher resolution images for Figure 7 would allow stronger conclusions.

Response: We agree with the reviewer that higher resolution images are needed to justify stronger conclusions. CryoET reconstructions of the EBV NEC coats are the focus of our future work, beyond the scope of the current study. In the meantime, we have toned down our interpretation.

**Reviewer #3:**

**Summary**

The revised manuscript addresses my earlier comments and adds new cryo-EM data that more directly address the oligomeric state of the EBV NEC, indicating that it does not adopt the hexameric form found in other herpesviruses. This data is consistent with the crystallographic structures and indicates plasticity in the assembly of herpesvirus NECS into larger oligomeric lattices on membranes. While the new data do not support the previous conclusion that the EBV NEC forms a conserved hexameric assembly, the mutational and structural studies support the conclusion that herpesvirus NECS utilize similar interfaces to mediate budding.

Response: We agree that the new cryo-EM data does not support the previous conclusion that the EBV NEC oligomerizes into a hexagonal lattice and thank the reviewer for their earlier suggestion.
